# The combined effect of policy changes and the covid-19 pandemic on the same day discharge and complications following total hip arthroplasty: a nationwide analysis

**DOI:** 10.1186/s42836-022-00131-w

**Published:** 2022-08-01

**Authors:** Adam M. Gordon, Matthew L. Magruder, Mitchell K. Ng, Bhavya K. Sheth, Charles A. Conway, Che Hang Jason Wong

**Affiliations:** Maimonides Medical Center, Department of Orthopaedic Surgery, 927 49th Street, Brooklyn New York, 11219 USA

**Keywords:** NSQIP, Orthopedics, Complications, Total hip arthroplasty, Covid-19, Elective surgery, Same day discharge

## Abstract

**Introduction:**

As a result of the SARS-CoV-2 (COVID-19) pandemic in 2020, elective surgeries, including total joint arthroplasty (TJA), were suspended nationwide. Concurrent removal of total hip arthroplasty (THA) from the Medicare inpatient-only list posed challenges to the delivery of quality patient care with low payor cost. Therefore, the objective of this study was to compare temporal trends in patient demographics, case volumes, length of stay, and complications following elective THA in the years 2019 to 2020 in the United States.

**Methods:**

The 2019 to 2020 ACS-NSQIP database was queried for elective THA patients. Patients Pre-COVID (2019 and 2020Q1) were compared with post-COVID (2020Q2-Q4). THA utilization, demographics, 30-day complications, and length of stay (LOS) were compared between years. Linear regression evaluated changes in case volumes over time with significance threshold of *P* < 0.05.

**Results:**

A total of 77,797 patients underwent elective THA in 2019 (*n* = 43,667) and 2020 (*n* = 34,130), resulting in a 24.5% decline. Outpatient THA increased in 2020 (35.6%) *vs*. 2019 (5.7%) (*P* < 0.001). There was no significant difference in the volume of cases in 2019Q1 through 2019Q4 (*P* = 0.984). Elective THA volumes declined by 68.8% in 2020Q2, returned to pre-pandemic baseline in 2020Q3, before leveling off at 81.5% of baseline in Q4. Average LOS was significantly shorter in 2020 (1.55 days) *vs*. 2019 (1.78 days) (*P* < 0.001) and the proportion of same day discharge (SDD) increased quarterly from 2019 to 2020. There was no significant difference in the total complication rates in 2019 (6.6%) *vs*. 2020 (6.6%) (*P* = 0.831).

**Discussion:**

Elective THA precipitously declined during the second quarter of 2020. The combined effect of policy changes and the COVID-19 pandemic resulted in a seven-fold increase in the number of surgeries performed in the outpatient setting in 2020. Rates of SDD doubled over the study period, despite no change in complication rates.

## Introduction

The SARS-CoV-2 (COVID-19) pandemic was first reported in the United States on January 30^th^, 2020 [1]. In order to contain the spread of disease and allocate healthcare workers and resources appropriately, elective surgery including total joint arthroplasty (TJA) was suspended nationwide [2]. On March 10, 2020, the World Health Organization (WHO) declared COVID-19 a worldwide pandemic [3]. Subsequently, the Surgeon General and the Centers for Medicare & Medicaid Services (CMS) declared the cancellation of all elective surgery in the United States in March 2020 [2].

The ramifications of cancelling elective joint arthroplasty have been felt by both patients and orthopedic joint replacement surgeons in the United States [4–13]. From a surgeon’s perspective, the projected backlog of cases was estimated to take between 9 to 35 months to recover [14]. Equally as concerning, the number of patients defined to be in a health quality of life state "worse than death" (WTD) due to waiting for a total joint replacement doubled during the pandemic [5]. Current total joint arthroplasty studies evaluating the decline in case volume due to COVID-19 are limited by lack of sample size and annual trend analysis. The few studies on this topic are either from countries outside the United States, review articles, single institutional evaluations, small multicenter collaborations, or simple projections based on historical data [4, 14–19]. Lowry Barnes *et al*. analyzed Medicare Claims limited to the first quarter of 2020. They reported a steep decline in TKA and THA volumes in mid-March of 94% and 92% [4]. More importantly, the clinical outcomes and complications for patients undergoing THA during the pandemic have not been reported.

In light of the worldwide suspension of elective total joint arthroplasty and the lack of nationwide reporting on an adequate representative sample, the primary purpose of the current study was to compare temporal trends in case volume of elective total hip arthroplasty (THA) from 2019 (pre-pandemic) to 2020 in the United States using a nationwide database. Secondarily, we sought to compare patient demographics, length of stay, and postoperative outcomes of those undergoing surgery before and after the pandemic origin. We hypothesized that not only would case volumes precipitously decline in 2020, but patient demographics and postoperative outcomes may be different.

## Materials and methods

### Database and patient selection

This study is a retrospective analysis of prospectively collected data from the 2019 to 2020 American College of Surgeons National Surgery Quality Improvement Program (ACS-NSQIP) database [20–24]. The NSQIP database includes detailed patient demographics in addition to preoperative and 30-day postoperative outcomes on patients undergoing major surgery. As of 2019, the database contained over 1 million cases from 719 participating institutions in the United States. The database is a source of accurate data, directly recording in-hospital morbidity and mortality as well as 30-day complications.

The database was queried for all patients undergoing elective THA (Current Procedural Terminology code 27130) in 2019 and 2020. Non-elective cases were excluded. Patients undergoing revision THA or conversion THA were also excluded from the study. Cases involving polytrauma, malignancies, or infections were excluded by using International Classification of Diseases, 9 or 10 Revision codes. Patients with missing demographic data were omitted from the study. As the data were derived from a de-identified national surgical database, the study was therefore exempt from Institutional Review Board (IRB) approval.

### Variables and outcomes studied

The change in national case volume from 2019 to 2020 was investigated. Secondarily, we directly compared admission quarters to evaluate the true influence of the COVID-19 pandemic on THA utilization over time. As admission quarter 1 (Q1) ends March 31, a comparison in the year prior to (2019) and during (2020) the COVID-19 pandemic was performed.

Patient demographics, included as part of the study, were age, gender, race, body mass index (BMI), and comorbidities (diabetes mellitus, smoking history, chronic obstructive pulmonary disease, congestive heart failure, hypertension, dialysis-dependent, disseminated cancer, chronic steroid use, bleeding disorder, ascites, dyspnea, and functional health status). The 5-item modified frailty index (mFI-5) was calculated for each patient by assigning one point for each comorbidity present: diabetes mellitus, hypertension, congestive heart failure, chronic obstructive pulmonary disease, and functionally dependent health status [25–28]. Operative and postoperative data included American Society of Anesthesiologists (ASA) grade, anesthesia administered, total operative time and length of stay (LOS) [29].

### Postoperative complications

Short-term postoperative complications (medical and surgical) were recorded and grouped into major and minor complications. Major complications included deep infections, organ infections, unplanned intubations, pulmonary emboli, ventilator use > 48 h, strokes, cardiac arrests, deep vein thromboses, sepsis, acute renal failures, blood transfusions, return to the operating room, and death. Complications were further broken down into the following broad categories: infection (superficial or deep surgical site infection), wound (wound dehiscence or other complications, not including surgical site infection), cardiac (cardiac arrest or myocardial infarction), pulmonary (pneumonia, pulmonary embolism, unplanned reintubation), hematological (deep vein thromboembolism, need for transfusion), renal (progressive renal insufficiency, acute kidney failure) issues, and adverse hospital discharge (discharge to other than home). Clavien Dindo IV complications (life-threatening complications including cardiac arrest, myocardial infarction, septic shock, pulmonary embolism, and renal failure) were collected and analyzed separately [30]. Rates of 30-day complications, reoperations, and readmissions were evaluated annually.

### Statistical analyses

Bivariate analysis using Pearson chi-squared tests, student’s *t* test, and analysis of variance (ANOVA) were used to assess the differences in patient demographics between years and admission quarters. Linear regression was used to evaluate for changes in the case volume over the study period. A statistical significance threshold of *P* < 0.05 was used. Statistical analysis was performed using SPSS version 24 [International Business Machine (IBM), Armonk, NY, USA)].

## Results

### Patient demographics

A total of 77,797 patients underwent elective THA in 2019 (*n* = 43,667) and 2020 (*n* = 34,130) (Table 1). The majority of patients were white, female, with an ASA class 2 comorbidity burden. Patient demographics of 2019 *vs*. 2020 cohorts were similar with respect to age, gender, BMI, and the presence of the following comorbidities (diabetes mellitus, tobacco use, chronic obstructive pulmonary disease, heart failure, hypertension). A significant increase was noted in the number of patients who underwent outpatient THA in 2020 *vs*. 2019 (35.6% *vs*. 5.7%; *P* < 0.001). Further breakdown comparing 2019 and 2020Q1 *vs*. 2020Q2-Q4 demonstrated that patients undergoing elective surgery during the COVID pandemic (after 2020Q2) were younger (65.5 years *vs*. 65.8 years; *P* = 0.003) and stayed in the hospital shorter (1.50 days *vs*. 1.76 days; *P* < 0.001) (Table 2). BMI was similar between cohorts (30.4 *vs*. 30.5; *P* = 0.127).

**Table 1:** Comparison of patient demographics in 2019 versus 2020 for elective THA

		2019		2020		*P* Value
		# Number of Patients	%	# Number of Patients	%	
**Number of Patients**		43,667		34,130		
**Age Cohorts**	< 40	869	2.00%	695	2.00%	0.122
	40–44	774	1.80%	611	1.80%	
	45–49	1558	3.60%	1141	3.30%	
	50–54	2969	6.80%	2376	7.00%	
	55–59	5374	12.30%	4212	12.30%	
	60–64	7391	16.90%	5756	16.90%	
	65–69	7969	18.20%	6489	19.00%	
	70–74	7440	17.00%	5797	17.00%	
	75–79	4987	11.40%	3794	11.10%	
	80–84	2817	6.50%	2081	6.10%	
	85 +	1519	3.50%	1178	3.50%	
**Gender**	female	23,742	54.40%	18,368	53.80%	0.299
	male	19,925	45.60%	15,762	46.20%	
**Race**	American Indian or Alaska Native	163	0.40%	152	0.40%	< 0.001
	Asian	594	1.40%	709	2.10%	
	Black or African American	3636	8.30%	3013	8.80%	
	Native Hawaiian or Pacific Islander	74	0.20%	75	0.20%	
	Some Other Race	0	0.00%	32	0.10%	
	Unknown/Not Reported	10,442	23.90%	7339	21.50%	
	White	28,758	65.90%	22,810	66.80%	
**BMI Category**	< 18.5	331	0.80%	290	0.90%	0.241
	18.5–24.9	7648	17.60%	6129	18.10%	
	25.0–29.9	14,465	33.30%	11,092	32.80%	
	30.0–34.9	11,635	26.80%	9078	26.80%	
	35.0–39.9	6233	14.30%	4871	14.40%	
	40.0 +	3124	7.20%	2399	7.10%	
**Diabetes mellitus**	Insulin Dependent	1282	2.90%	966	2.80%	0.632
	No	38,263	87.60%	29,910	87.60%	
	Non-Insulin Dependent	4122	9.40%	3254	9.50%	
**Current smoker**	No	38,621	88.40%	30,291	88.80%	0.181
	Yes	5046	11.60%	3839	11.20%	
**Dyspnea**	At Rest	52	0.10%	37	0.10%	0.007
	Moderate Exertion	2001	4.60%	1405	4.10%	
	No	41,614	95.30%	32,688	95.80%	
**Functional health status**	Independent	42,755	97.90%	33,431	98.00%	< 0.001
	Partially Dependent	570	1.30%	557	1.60%	
	Totally Dependent	25	0.10%	37	0.10%	
	Unknown	317	0.70%	105	0.30%	
**History of Severe COPD**	No	42,026	96.20%	32,923	96.50%	0.103
	Yes	1641	3.80%	1207	3.50%	
**Congestive heart failure (CHF)**	No	43,483	99.60%	33,986	99.60%	0.991
	Yes	184	0.40%	144	0.40%	
**Hypertension**	No	19,743	45.20%	15,543	45.50%	0.362
	Yes	23,924	54.80%	18,587	54.50%	
**Currently on dialysis (pre-op)**	No	43,588	99.80%	34,057	99.80%	0.301
	Yes	79	0.20%	73	0.20%	
**Steroid use for chronic condition**	No	42,170	96.60%	32,955	96.60%	0.912
	Yes	1497	3.40%	1175	3.40%	
**> 10% loss body weight in last 6 months**	No	43,583	99.80%	34,053	99.80%	0.311
	Yes	84	0.20%	77	0.20%	
**Bleeding disorders**	No	42,917	98.30%	33,491	98.10%	0.106
	Yes	750	1.70%	639	1.90%	
**mFI**	0	18,162	41.60%	14,278	41.80%	0.625
	1	19,707	45.10%	15,309	44.90%	
	2	5384	12.30%	4205	12.30%	
	3	386	0.90%	319	0.90%	
	4	25	0.10%	19	0.10%	
	5	3	0.00%	0	0.00%	
**Inpatient/Outpatient**	Inpatient	41,162	94.30%	21,977	64.40%	< 0.001
	Outpatient	2505	5.70%	12,153	35.60%	
**ASA classification**	1-No Disturbance	1419	3.20%	1004	2.90%	0.012
	2-Mild Disturbance	22,179	50.80%	17,310	50.70%	
	3-Severe Disturbance	19,273	44.10%	15,101	44.20%	
	4-Life Threat	759	1.70%	690	2.00%	f
**Principal anesthesia technique**	Epidural	266	0.60%	222	0.70%	< 0.001
	General	17,782	40.70%	13,149	38.50%	
	MAC/IV Sedation	8963	20.50%	7962	23.30%	
	Regional	509	1.20%	663	1.90%	
	Spinal	16,111	36.90%	12,109	35.50%	
	Other	36	0.08%	25	0.07%	
**Total operation time (minutes)**		89.81		91.65		< 0.001
**Length of hospital stay (days)**		1.78		1.55		< 0.001

**Table 2:** Comparison of patient demographics per quarter for elective THA

**Quarter of Operation**		**2019**				**2020**				***P* Value**
		**1**	**2**	**3**	**4**	**1**	**2**	**3**	**4**	
**Number of Patients**		10,591	11,360	10,987	10,729	9448	5331	10,451	8900	
**Age Cohorts**	< 40	204 (1.9)	217 (1.9)	208 (1.9)	240 (2.2)	185 (2.0)	124 (2.3)	207 (2.0)	179 (2.0)	< 0.001
	40–44	178 (1.7)	209 (1.8)	188 (1.7)	199 (1.9)	171 (1.8)	92 (1.7)	174 (1.7)	174 (2.0)	
	45–49	377 (3.6)	387 (3.4)	374 (3.4)	420 (3.9)	294 (3.1)	184 (3.5)	325 (3.1)	338 (3.8)	
	50–54	720 (6.8)	762 (6.7)	745 (6.8)	742 (6.9)	637 (6.7)	396 (7.4)	676 (6.5)	667 (7.5)	
	55–59	1302 (12.3)	1386 (12.2)	1293 (11.8)	1393 (13.0)	1098 (11.6)	753 (14.1)	1220 (11.7)	1141 (12.8)	
	60–64	1785 (16.9)	1927 (17.0)	1809 (16.5)	1870 (17.4)	1550 (16.4)	868 (16.3)	1771 (16.9)	1567 (17.6)	
	65–69	1968 (18.6)	2059 (18.1)	2016 (18.3)	1926 (18.0)	1869 (19.8)	1007 (18.9)	1917 (18.3)	1696 (19.1)	
	70–74	1817 (17.2)	1929 (17.0)	1966 (17.9)	1728 (16.1)	1586 (16.8)	904 (17.0)	1870 (17.9)	1437 (16.1)	
	75–79	1167 (11.0)	1289 (11.3)	1295 (11.8)	1236 (11.5)	1095 (11.6)	539 (10.1)	1230 (11.8)	930 (10.4)	
	80–84	702 (6.6)	780 (6.9)	706 (6.4)	629 (5.9)	582 (6.2)	308 (5.8)	679 (6.5)	512 (5.8)	
	85 +	371 (3.5)	415 (3.7)	387 (3.5)	346 (3.2)	381 (4.0)	156 (2.9)	382 (3.7)	259 (2.9)	
**Gender**	female	5610 (53.0)	6322 (55.7)	6159 (56.1)	5651 (52.7)	5090 (53.9)	2863 (53.7)	5716 (54.7)	4699 (52.8)	< 0.001
	male	4981 (47.0)	5038 (44.3)	4828 (43.9)	5078 (47.3)	4358 (46.1)	2468 (46.3)	4735 (45.3)	4201 (47.2)	
**Race**	American Indian or Alaska Native	38 (.4)	41 (.4)	40 (.4)	44 (.4)	42 (.4)	33 (.6)	38 (.4)	39 (.4)	< 0.001
	Asian	140 (1.3)	169 (1.5)	141 (1.3)	144 (1.3)	133 (1.4)	121 (2.3)	229 (2.2)	226 (2.5)	
	Black or African American	833 (7.9)	968 (8.5)	946 (8.6)	889 (8.3)	809 (8.6)	405 (7.6)	955 (9.1)	844 (9.5)	
	Native Hawaiian or Pacific Islander	20 (.2)	16 (.1)	23 (.2)	15 (.1)	33 (.3)	12 (.2)	16 (.2)	14 (.2)	
	Some Other Race	0 (.0)	0 (.0)	0 (.0)	0 (.0)	2 (.0)	0 (.0)	10 (.1)	20 (.2)	
	Unknown/Not Reported	2538 (24.0)	2662 (23.4)	2579 (23.5)	2663 (24.8)	2156 (22.8)	795 (14.9)	2326 (22.3)	2062 (23.2)	
	White	7022 (66.3)	7504 (66.1)	7258 (66.1)	6974 (65.0)	6273 (66.4)	3965 (74.4)	6877 (65.8)	5695 (64.0)	
**BMI Category**	< 18.5	70 (.7)	99 (.9)	87 (.8)	75 (.7)	77 (.8)	37 (.7)	93 (.9)	83 (.9)	0.444
	18.5–24.9	1882 (17.8)	2022 (17.8)	1883 (17.1)	1861 (17.3)	1633 (17.3)	972 (18.2)	1913 (18.3)	1611 (18.1)	
	25.0–29.9	3542 (33.4)	3682 (32.4)	3682 (33.5)	3559 (33.2)	3054 (32.3)	1742 (32.7)	3400 (32.5)	2896 (32.5)	
	30.0–34.9	2811 (26.5)	3005 (26.5)	2925 (26.6)	2894 (27.0)	2611 (27.6)	1424 (26.7)	2687 (25.7)	2356 (26.5)	
	35.0–39.9	1473 (13.9)	1671 (14.7)	1562 (14.2)	1527 (14.2)	1377 (14.6)	721 (13.5)	1508 (14.4)	1265 (14.2)	
	40.0 +	756 (7.1)	823 (7.2)	785 (7.1)	760 (7.1)	650 (6.9)	373 (7.0)	757 (7.2)	619 (7.0)	
**Diabetes mellitus**	Insulin Dependent	298 (2.8)	318 (2.8)	340 (3.1)	326 (3.0)	264 (2.8)	136 (2.6)	319 (3.1)	247 (2.8)	0.389
	No	9303 (87.8)	9928 (87.4)	9625 (87.6)	9407 (87.7)	8304 (87.9)	4720 (88.5)	9121 (87.3)	7765 (87.2)	
	Non-Insulin Dependent	990 (9.3)	1114 (9.8)	1022 (9.3)	996 (9.3)	880 (9.3)	475 (8.9)	1011 (9.7)	888 (10.0)	
**Current smoker**	No	9346 (88.2)	10,069 (88.6)	9669 (88.0)	9537 (88.9)	8374 (88.6)	4785 (89.8)	9270 (88.7)	7862 (88.3)	0.054
	Yes	1245 (11.8)	1291 (11.4)	1318 (12.0)	1192 (11.1)	1074 (11.4)	546 (10.2)	1181 (11.3)	1038 (11.7)	
**Dyspnea**	At Rest	15 (.1)	13 (.1)	12 (.1)	12 (.1)	9 (.1)	6 (.1)	11 (.1)	11 (.1)	0.007
	Moderate Exertion	488 (4.6)	514 (4.5)	519 (4.7)	480 (4.5)	406 (4.3)	161 (3.0)	446 (4.3)	392 (4.4)	
	No	10,088 (95.3)	10,833 (95.4)	10,456 (95.2)	10,237 (95.4)	9033 (95.6)	5164 (96.9)	9994 (95.6)	8497 (95.5)	
**Functional health status**	Independent	10,380 (98.0)	11,105 (97.8)	10,744 (97.8)	10,526 (98.1)	9264 (98.1)	5244 (98.4)	10,230 (97.9)	8693 (97.7)	< 0.001
	Partially Dependent	144 (1.4)	153 (1.3)	139 (1.3)	134 (1.2)	136 (1.4)	73 (1.4)	190 (1.8)	158 (1.8)	
	Totally Dependent	5 (.0)	7 (.1)	8 (.1)	5 (.0)	5 (.1)	7 (.1)	12 (.1)	13 (.1)	
	Unknown	62 (.6)	95 (.8)	96 (.9)	64 (.6)	43 (.5)	7 (.1)	19 (.2)	36 (.4)	
**History of Severe COPD**	No	10,214 (96.4)	10,938 (96.3)	10,538 (95.9)	10,336 (96.3)	9127 (96.6)	5168 (96.9)	10,019 (95.9)	8609 (96.7)	0.001
	Yes	377 (3.6)	422 (3.7)	449 (4.1)	393 (3.7)	321 (3.4)	163 (3.1)	432 (4.1)	291 (3.3)	
**Congestive heart failure (CHF)**	No	10,556 (99.7)	11,297 (99.4)	10,947 (99.6)	10,683 (99.6)	9408 (99.6)	5311 (99.6)	10,401 (99.5)	8866 (99.6)	0.241
	Yes	35 (.3)	63 (.6)	40 (.4)	46 (.4)	40 (.4)	20 (.4)	50 (.5)	34 (.4)	
**Hypertension**	No	4870 (46.0)	5044 (44.4)	4852 (44.2)	4977 (46.4)	4306 (45.6)	2456 (46.1)	4702 (45.0)	4079 (45.8)	0.007
	Yes	5721 (54.0)	6316 (55.6)	6135 (55.8)	5752 (53.6)	5142 (54.4)	2875 (53.9)	5749 (55.0)	4821 (54.2)	
**Currently on dialysis (pre-op)**	No	10,575 (99.8)	11,338 (99.8)	10,966 (99.8)	10,709 (99.8)	9431 (99.8)	5317 (99.7)	10,432 (99.8)	8877 (99.7)	0.734
	Yes	16 (.2)	22 (.2)	21 (.2)	20 (.2)	17 (.2)	14 (.3)	19 (.2)	23 (.3)	
**Steroid use for chronic condition**	No	10,221 (96.5)	10,978 (96.6)	10,605 (96.5)	10,366 (96.6)	9127 (96.6)	5155 (96.7)	10,081 (96.5)	8592 (96.5)	0.993
	Yes	370 (3.5)	382 (3.4)	382 (3.5)	363 (3.4)	321 (3.4)	176 (3.3)	370 (3.5)	308 (3.5)	
** > 10% loss body weight in last 6 months**	No	10,574 (99.8)	11,337 (99.8)	10,965 (99.8)	10,707 (99.8)	9428 (99.8)	5316 (99.7)	10,425 (99.8)	8884 (99.8)	0.804
	Yes	17 (.2)	23 (.2)	22 (.2)	22 (.2)	20 (.2)	15 (.3)	26 (.2)	16 (.2)	
**Bleeding disorders**	No	10,438 (98.6)	11,170 (98.3)	10,772 (98.0)	10,537 (98.2)	9274 (98.2)	5235 (98.2)	10,243 (98.0)	8739 (98.2)	0.088
	Yes	153 (1.4)	190 (1.7)	215 (2.0)	192 (1.8)	174 (1.8)	96 (1.8)	208 (2.0)	161 (1.8)	
**mFI**	0	4499 (42.5)	4637 (40.8)	4469 (40.7)	4557 (42.5)	3964 (42.0)	2269 (42.6)	4312 (41.3)	3733 (41.9)	0.013
	1	4715 (44.5)	5171 (45.5)	5028 (45.8)	4793 (44.7)	4261 (45.1)	2415 (45.3)	4651 (44.5)	3982 (44.7)	
	2	1285 (12.1)	1442 (12.7)	1373 (12.5)	1284 (12.0)	1144 (12.1)	609 (11.4)	1359 (13.0)	1093 (12.3)	
	3	84 (.8)	102 (.9)	109 (1.0)	91 (.8)	77 (.8)	36 (.7)	122 (1.2)	84 (.9)	
	4	7 (.1)	8 (.1)	8 (.1)	2 (.0)	2 (.0)	2 (.0)	7 (.1)	8 (.1)	
	5	1 (.0)	0 (.0)	0 (.0)	2 (.0)	0 (.0)	0 (.0)	0 (.0)	0 (.0)	
**Inpatient/outpatient**	Inpatient	10,119 (95.5)	10,756 (94.7)	10,336 (94.1)	9951 (92.7)	7249 (76.7)	3273 (61.4)	6380 (61.0)	5075 (57.0)	< 0.001
	Outpatient	472 (4.5)	604 (5.3)	651 (5.9)	778 (7.3)	2199 (23.3)	2058 (38.6)	4071 (39.0)	3825 (43.0)	
**ASA classification**	1-No Disturbance	354 (3.3)	374 (3.3)	311 (2.8)	380 (3.5)	291 (3.1)	155 (2.9)	270 (2.6)	288 (3.2)	< 0.001
	2-Mild Disturbance	5417 (51.1)	5711 (50.3)	5591 (50.9)	5460 (50.9)	4766 (50.4)	2849 (53.4)	5113 (48.9)	4582 (51.5)	
	3-Severe Disturbance	4655 (44.0)	5031 (44.3)	4872 (44.3)	4715 (43.9)	4195 (44.4)	2233 (41.9)	4825 (46.2)	3848 (43.2)	
	4-Life Threat	155 (1.5)	231 (2.0)	202 (1.8)	171 (1.6)	194 (2.1)	94 (1.8)	234 (2.2)	168 (1.9)	
**Principal anesthesia technique**	Epidural	53 (.5)	65 (.6)	69 (.6)	79 (.7)	42 (.4)	47 (.9)	90 (.9)	43 (.5)	< 0.001
	General	4366 (41.2)	4584 (40.4)	4501 (41.0)	4331 (40.4)	3689 (39.0)	2171 (40.7)	3981 (38.1)	3308 (37.2)	
	MAC/IV Sedation	2173 (20.5)	2287 (20.1)	2283 (20.8)	2220 (20.7)	2202 (23.3)	1133 (21.3)	2465 (23.6)	2162 (24.3)	
	Regional	110 (1.0)	127 (1.1)	131 (1.2)	141 (1.3)	162 (1.7)	116 (2.2)	210 (2.0)	175 (2.0)	
	Spinal	3879 (36.6)	4287 (37.7)	3999 (36.4)	3946 (36.8)	3346 (35.4)	1859 (34.9)	3698 (35.4)	3206 (36.0)	
	Other	10 (0.1)	10 (0.1)	4 (0.0)	12 (0.1)	7 (0.1)	5 (0.1)	7 (0.1)	6 (0.1)	
**Total operation time (minutes)**		89.37	89.48	89.99	90.4	90.59	91.93	91.85	92.39	< 0.001
**Length of hospital stay (days)**		1.86	1.79	1.76	1.68	1.68	1.48	1.52	1.48	< 0.001
**Length of Stay (days)**	0	899 (8.5)	1066 (9.4)	1072 (9.8)	1195 (11.1)	1057 (11.2)	1074 (20.1)	1869 (17.9)	1854 (20.8)	< 0.001
	1	4518 (42.7)	4969 (43.7)	4997 (45.5)	5081 (47.4)	4746 (50.2)	2686 (50.4)	5245 (50.2)	4435 (49.8)	
	≥ 2	5158 (48.7)	5320 (46.8)	4912 (44.7)	4450 (41.5)	3637 (38.5)	1571 (29.5)	3336 (31.9)	2609 (29.3)	

Values are reported as N (%) or means

*mFI* Modified Frailty Index

### Quarterly trends in THA utilization

**Fig. 1:**
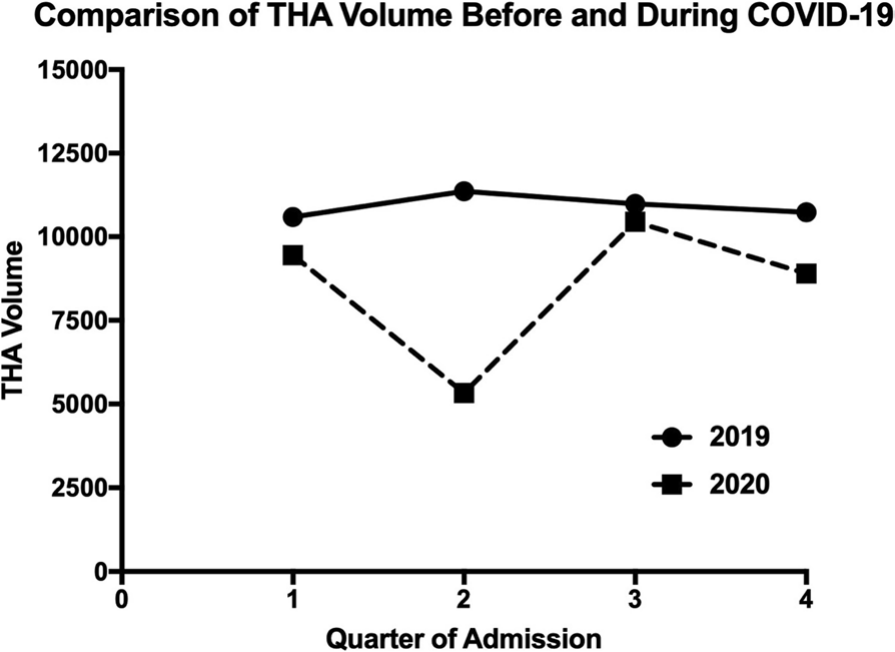
Nationwide comparison of elective THA volume by quarter. There was no significant difference in the volume of cases in 2019Q1 through 2019Q4 (*p* = 0.984)**.** However, compared to 2019, elective THA volumes minimally declined by 14.4% in 2020Q1, and drastically declined by 68.8% in 2020Q2. Elective THA case volumes returned to pre-pandemic baseline in 2020Q3 before eventually leveling off at 81.5% of baseline

Overall, there was a 24.5% decline in elective THA from 2019 to 2020. There was no significant difference in the volume of cases in 2019Q1 through 2019Q4 (*P* = 0.984) (Fig. 1). However, compared to 2019, elective THA volumes minimally dropped by 14.4% in 2020Q1, and drastically decreased by 68.8% in 2020Q2 (Fig. 1). Elective THA case volumes returned to pre-pandemic baseline in 2020Q3 before eventually leveling off at 81.5% of baseline (Fig. 1).

### Postoperative outcomes and complications

**Fig. 2:**
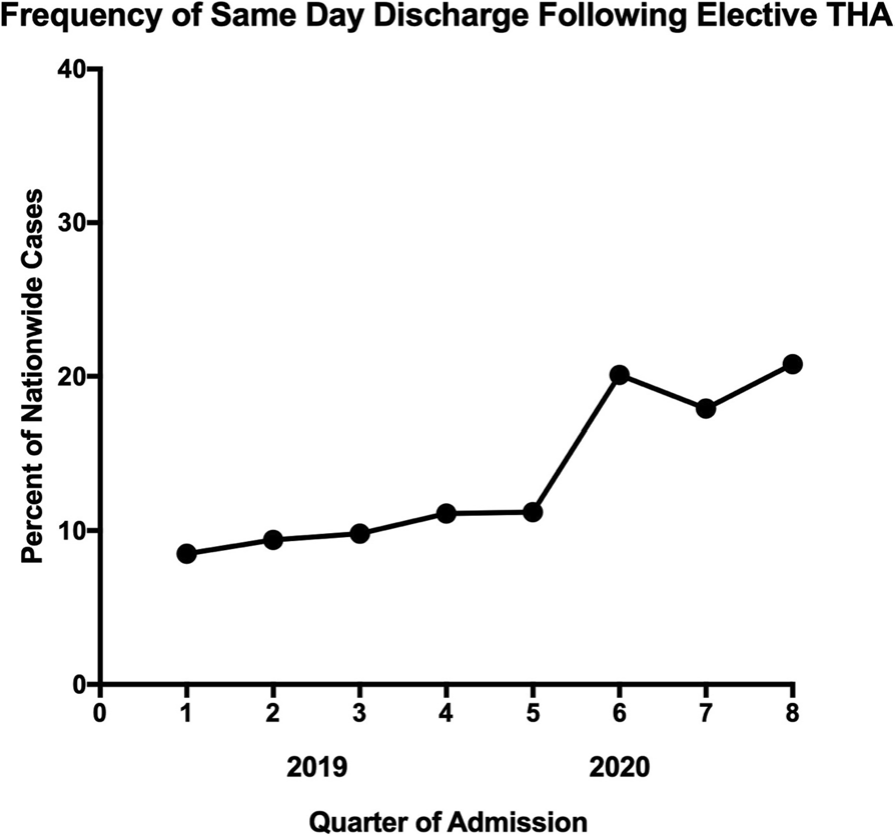
The proportion of same day discharge increased by quarter from 2019 (Ranging from 8.5% to 11.1%) to 2020 (Ranging from 11.2% to 20.8%)

The average length of stay was significantly shorter in 2020 *vs*. 2019 (1.55 days *vs*. 1.78 days; *P* < 0.001). The proportion of same day discharge increased quarterly from 2019 to 2020 (Fig. 2). The overall 30-day complication rate was 6.6% (5,112/77,797). There was no significant difference in the total complication rates in 2019 (6.6%) *vs*. 2020 (6.6%) (*P* = 0.831). When comparing 2019 to 2020, rates of major complications (5.2% *vs*. 5.1%; *P* = 0.944), infection (1.3% *vs*. 1.4%; *P* = 0.415), wound complications (0.16% *vs*. 0.17%; *P* = 0.669), cardiac (0.24% *vs*. 0.29%; *P* = 0.205), pulmonary (0.56% *vs*. 0.53%; *P* = 0.584), hematological (3.0% *vs*. 3.0%; *P* = 0.801), renal complications (0.09% *vs*. 0.12%; *P* = 0.183), and Clavien Dindo IV complications (0.55% *vs*. 0.54%; *P* = 0.965) were similar. The overall 30-day mortality was significantly higher in 2020 (0.15%) *vs*. 2019 (0.09%); *P* = 0.011. Thirty-day reoperation (1.75% *vs.* 1.62%; *P* = 0.175) and readmission rates (3.16% vs 2.93%; *p* = 0.066) were no different between calendar years.

## Discussion

Total hip arthroplasty (THA) and total knee arthroplasty (TKA) are some of the highest-volume procedures performed in hospitals on an elective basis in the United States. The effects of the COVID-19 pandemic on elective total hip arthroplasty case volumes in the United States are still being investigated [31]. To date, an adequate nationwide representation of joint arthroplasty decline in the calendar year 2020 has yet to be reported. Here we presented the first temporal trends analysis of elective total hip arthroplasty in the year prior to and during the COVID-19 pandemic. In this study, we found a 24.5% decline in annual elective THA from 2019 to 2020. The volume of cases in 2019Q1 through 2019Q4 remained constant, with a significant 68.8% decline in 2020Q2. These results confirmed our hypothesis that in quarter 2 of 2020, the effects of the pandemic-related restrictions on elective surgery would be apparent. Unknown to the orthopedic arthroplasty community was whether case volumes would recover or remain lower than the prior year. The combined effect of policy changes and the COVID-19 pandemic resulted in a seven-fold increase in the number of surgeries performed in the outpatient setting in 2020, with no change in complication rates.

TJA is responsible for a significant amount of revenue for the health care system but is also viewed as a non-essential procedure during the COVID-19 pandemic. Comparing annual elective THA, we found a 24.5% decline from 2019 to 2020. From 2019Q1 through 2019Q4 there was no significant difference in the volume of elective THA cases (*P* = 0.984). However, compared to 2019, elective THA volumes declined by 14.4% in 2020Q1, and drastically dropped by 68.8% in 2020Q2. Elective THA case volumes returned to pre-pandemic baseline in 2020Q3 before eventually plateauing at 81.5% of baseline. The results of our study mirror survey responses by American Association of Hip and Knee Surgeons (AAHKS) members related to their clinical practice during this time. The proportion of AAHKS members who reported a dramatic reduction of performing inpatient arthroplasty included 82% in late March 2020, peaking to 92% early April 2020, and then correcting to a 23% reduction by mid-June 2020 [11]. The consequences of this included a projected backlog of cases estimated to take 9 to 35 months to recover [14].

To date, no studies have evaluated the temporal trends in patient demographics of those undergoing elective surgery in the year prior to and during COVID-19. It was hypothesized that, nationwide, patients would be younger and healthier in order to promote minimal risk. Despite these predictions, we did not find any appreciable difference in annual cohorts with respect to age, BMI, and comorbidity burden. Groups have queried patients about their perceptions and feelings about delaying total joint arthroplasties during the pandemic [7, 8, 10, 13]. Patients have generally felt an increase in anxiety and decline in quality of life [5]. Although 85% of patients understood and agreed with the public health measures to curb infections, almost 90% of patients planned to reschedule their joint replacement as soon as possible [7].

Perhaps the most clinically important finding of our study was the notable shift of cases from the inpatient to outpatient setting before and during the pandemic. We found a seven-fold increase in outpatient THA cases. Furthermore, length of stay declined over each quarter of the study period, with frequencies of same day discharge doubling during the COVID-19 pandemic. The coinciding events including the onset of the COVID-19 pandemic and Centers for Medicare & Medicaid Services (CMS) removal of total hip arthroplasty from the “inpatient-only” list are responsible for this change [32–35]. In the present study, the overall 30-day complication rate was 6.6% (5,112/77,797). We found no significant differences in the major complications, infection, wound complications, cardiac, pulmonary, hematological, renal complications, and Clavien Dindo IV complications from 2019 to 2020. The 30-day reoperation (1.75% *vs*. 1.62%) and readmission (3.16% *vs*. 2.93%) rates following elective THA were low in our study and not impacted by the COVID-19 pandemic. However, the overall 30-day mortality was significantly higher in 2020 (0.15%) *vs*. 2019 (0.09%). While our data provided no direct cause of this mortality increase, it is probable that this was secondary to sequalae of the COVID-19 pandemic.

There are a few limitations to the study that warrant discussion with any national database evaluation of retrospectively collected data. The database used for this study is one of the largest nationwide representative samples, however, does not capture every hospital or surgery in the United States. Therefore, the case volume trends reported in this study should be taken in the appropriate context. Our inclusion criteria were narrowed to include only elective arthroplasty cases as this would ensure a homogeneous sample when comparing 2019 to 2020. The present study trends may be a result of other confounding factors, including changes in clinical practice instead of directly the COVID-19 pandemic. Data accuracy is potentially a concern, however, NSQIP undergoes auditing for inter-rater reliability to ensure the validity of the data [36]. All dependent variables of interest, including complications, reoperations, and readmissions, were limited to 30 days postoperatively, which do not capture patients who presented to the hospital after that time. The use of the ACS-NSQIP database prevented the ability to report on more granular postoperative outcomes such as the cause of mortality, the cause of reoperation, and exact cause of readmission. Despite these limitations, this is the first nationwide sample using this data to compare temporal trends in elective THA utilization prior to and during suspension of non-emergent surgery.

## Conclusion

In the United States, there was a 24.5% decline in elective THA in 2020. Case volumes precipitously declined by 68.8% during the second quarter of 2020, before returning to pre-pandemic baseline in 2020Q3 and eventually leveling off at 81.5% of baseline. Patient demographics of those undergoing elective THA in 2020 were similar in comorbidity burden. The combined effect of policy changes and the COVID-19 pandemic resulted in a seven-fold increase in the number of surgeries performed in the outpatient setting in 2020, with rates of same day discharge doubling over the study period.

## Data Availability

All data are publicly available to participating institutions. The ACS-NSQIP from which the data are derived have not verified and are not responsible for the statistical validity of the data analysis or the conclusions derived by the authors.

## Abbreviations

ACS-NSQIP: American College of Surgeons National Surgical Quality Improvement Program
ASA: American Society of Anesthesia
CMS: Centers for Medicare & Medicaid Services
COVID-19: Coronavirus Disease 2019
mFI: Modified Frailty Index
SDD: Same Day Discharge
THA: Total Hip Arthroplasty
TJA: Total Joint Arthroplasty
WHO: World Health Organization
